# Concordance between single-nucleotide polymorphism–based genomic instability assays and a next-generation sequencing–based homologous recombination deficiency test

**DOI:** 10.1186/s12885-022-10197-z

**Published:** 2022-12-14

**Authors:** Razvan Cristescu, Xiao Qiao Liu, Gladys Arreaza, Cai Chen, Andrew Albright, Ping Qiu, Matthew J. Marton

**Affiliations:** 1 Merck & Co., Inc, 90 E Scott Ave, Rahway, NJ USA; 2 MSD R&D (China) Co. Ltd, Beijing, China

**Keywords:** BRCA, Loss of heterozygosity, Homologous recombination deficiency, Large-scale state transition, Telomeric-allelic imbalance number

## Abstract

**Background:**

We evaluated the performance of single-nucleotide polymorphism (SNP) genotyping arrays OncoScan (Thermo Fisher Scientific, San Diego, CA) and Infinium CytoSNP-850K (CytoSNP; Illumina, Waltham, MA) for assessing homologous recombination deficiency (HRD) genomic instability.

**Methods:**

DNA (pretreatment samples) across 20 tumor types was evaluated with OncoScan, CytoSNP, and the clinically validated HRD test. Copy number variation (CNV) and loss of heterozygosity (LOH) analyses were performed with ASCATv2.5.1. Aggregate HRD genomic metrics included LOH, telomeric-allelic imbalance number (TAI), and large-scale state transition (LST). Associations between *BRCA* mutation (*BRCA*m) status and the clinically validated HRD test metric (dichotomized at a clinical cut-off) were evaluated using area under the receiver operating characteristic (AUROC); Spearman ρ was calculated for continuous metrics. CNV segmentation and HRD genomic metrics were calculated (*n* = 120, *n* = 106, and *n* = 126 for OncoScan, CytoSNP and clinically validated HRD test, respectively).

**Results:**

When assessed by SNP arrays, the genomic metric demonstrated good association with *BRCA*m (AUROC of HRD: OncoScan, 0.87; CytoSNP, 0.75) and the clinically validated test (cut-off, 42; AUROC of HRD: OncoScan, 0.92; CytoSNP, 0.91). The genomic metrics demonstrated good correlation with the clinically validated aggregate HRD test metric (ρ: OncoScan, 0.82; CytoSNP, 0.81) and for each component (ρ: OncoScan, 0.68 [LOH], 0.76 [TAI], and 0.78 [LST]; CytoSNP, 0.59 [LOH], 0.77 [TAI], and 0.82 [LST]). HRD assessed by SNP genotyping arrays and the clinically validated test showed good correlation.

**Conclusion:**

OncoScan and CytoSNP may potentially identify most HRD-positive tumors with appropriate clinically relevant cut-offs.

**Supplementary Information:**

The online version contains supplementary material available at 10.1186/s12885-022-10197-z.

## Background

Ovarian cancer remains a significant cause of mortality; options are needed in both first-line treatment and subsequent treatment for advanced disease [1]. Homologous recombination deficiency (HRD) is linked to germline or somatic mutations in *BRCA1* and/or *BRCA2* (*BRCA*m) as well as other genomic changes (i.e., epigenetic), and approximately half of all advanced high-grade serous ovarian cancers (HGSOCs) are HRD positive [1, 2]. Accordingly, treatment strategies aim to target this pathway, specifically the enzyme poly(adenosine diphosphate-ribose) polymerase (PARP). PARP inhibitors (PARPis) were developed with the knowledge of their direct antitumor activity and their effects on HGSOC cells harboring mutations in genes implicated in the homologous recombination repair pathway, including *BRCA*m [3]. Specifically, PARPis prevent efficient single-strand DNA repair activity, leading to genomic instability and cellular death in *BRCA*m or HRD-affected cells [1]. Thus, various biomarkers have been investigated to identify patients likely to respond to PARPis [4].

The combination of the PARPi olaparib and the vascular endothelial growth factor inhibitor bevacizumab is approved by the US Food and Drug Administration (FDA) as maintenance therapy for HRD-positive advanced ovarian cancer [5]. The FDA contemporaneously approved a companion diagnostic for HRD assessment based on a next-generation sequencing (NGS) assay that determines HRD status using assessments of *BRCA*m and genomic instability, which includes loss of heterozygosity (LOH), telomeric-allelic imbalance number (TAI), and large-scale state transition (LST) [6, 7]. Using this assay, tumors are considered HRD positive if they have a deleterious or suspected deleterious *BRCA*m or genomic instability defined as an HRD score of ≥ 42 or both [6]. HRD status or *BRCA*m status as determined by the test is prognostic of outcome [7]. Patients with ovarian cancer with either a germline or somatic deleterious or suspected deleterious *BRCA*m have better outcomes (i.e., improved response rates, progression-free survival, or overall survival) than patients with non–*BRCA*m tumors [8–12]. Additionally, patients with a high HRD score as determined by the test had a more favorable prognosis than patients with low HRD scores [9, 12–14]. The presence of a *BRCA*m or a high HRD score, or both, was also predictive of a better response to PARPis as monotherapy or in combination with bevacizumab [8, 11–13, 15].

Several tests are in development to assess HRD status; however, there is currently no standardized way to define, measure, or report HRD [16]. Further, many of these assays are complex, employing different approaches, data analysis algorithms, and cut-offs to assess HRD. This lack of harmonization across HRD assays highlights the importance of comparisons of available testing strategies; it could also introduce risk to patients if a clinical HRD test is reported or interpreted incorrectly relative to how HRD status has been clinically validated. Formal comparisons of available HRD assays are lacking, with the current study being one of the first few to investigate the utility of genomic instability score based on commercially available single-nucleotide polymorphism (SNP) arrays to assess HRD status in the support of PARPi use [17].

Genotyping microarrays that evaluate SNPs are among those being developed for HRD determination, which may provide an alternative to the NGS-based assay. SNP-based assays are designed to measure tumor-related copy number changes [9, 11, 18]. Herein, we evaluated the performance of two SNP-based assay platforms: OncoScan (Thermo Fisher Scientific, San Diego, CA) and Infinium CytoSNP-850K (Illumina, Waltham, MA), for assessing HRD genomic instability.

## Methods

### Clinical tumor samples

HRD was assessed from pretreatment archival tumor samples (formalin-fixed paraffin-embedded tissue) collected from clinical studies (KEYNOTE-001 [ClinicalTrials.gov Identifier: NCT01295827], KEYNOTE-012 [ClinicalTrials.gov Identifier: NCT01848834], KEYNOTE-028 [ClinicalTrials.gov Identifier: NCT02054806], KEYNOTE-055 [ClinicalTrials.gov Identifier: NCT02255097], KEYNOTE-061 [ClinicalTrials.gov Identifier: NCT02370498], KEYNOTE-086 [ClinicalTrials.gov Identifier: NCT02447003], KEYNOTE-100 [ClinicalTrials.gov Identifier: NCT02674061], and KEYNOTE-199 [ClinicalTrials.gov Identifier: NCT02787005]) funded by Merck Sharp & Dohme LLC, a subsidiary of Merck & Co., Inc., Rahway, NJ, USA, across various cancer types [19]. All patients provided written informed consent before enrollment in the clinical trials. All study protocols were consistent with the global standards of the International Conference on Harmonization Good Clinical Practices, the Council for International Organizations of Medical Sciences Public Policy Statement, Clinical Trial Ethics Sciences International Ethical Guidelines for Biomedical Research Involving Human Subjects (CIOMS, 2002), the Pharmaceutical Research and Manufacturers of America (PhRMA, 2009) Principles on Conduct of Clinical Trials, applicable local regulatory requirements, and the ethical principles that have their origin in the Declaration of Helsinki. All patient samples were analyzed anonymously and deidentified prior to use. The current analysis did not require IRB approval. All reference assay data were generated by Myriad Genetics at their Salt Lake City, UT, facility.

### DNA extraction

Specimens passing pathology review were queued for DNA extraction by lysing cells from formalin-fixed paraffin embedded tissue by digestion with a proteinase K buffer followed by automated purification using the 96-well KingFisher Flex Magnetic Particle Processor (Thermo Fisher Scientific, San Diego, CA) [20].

### Analyses

**Fig. 1:**
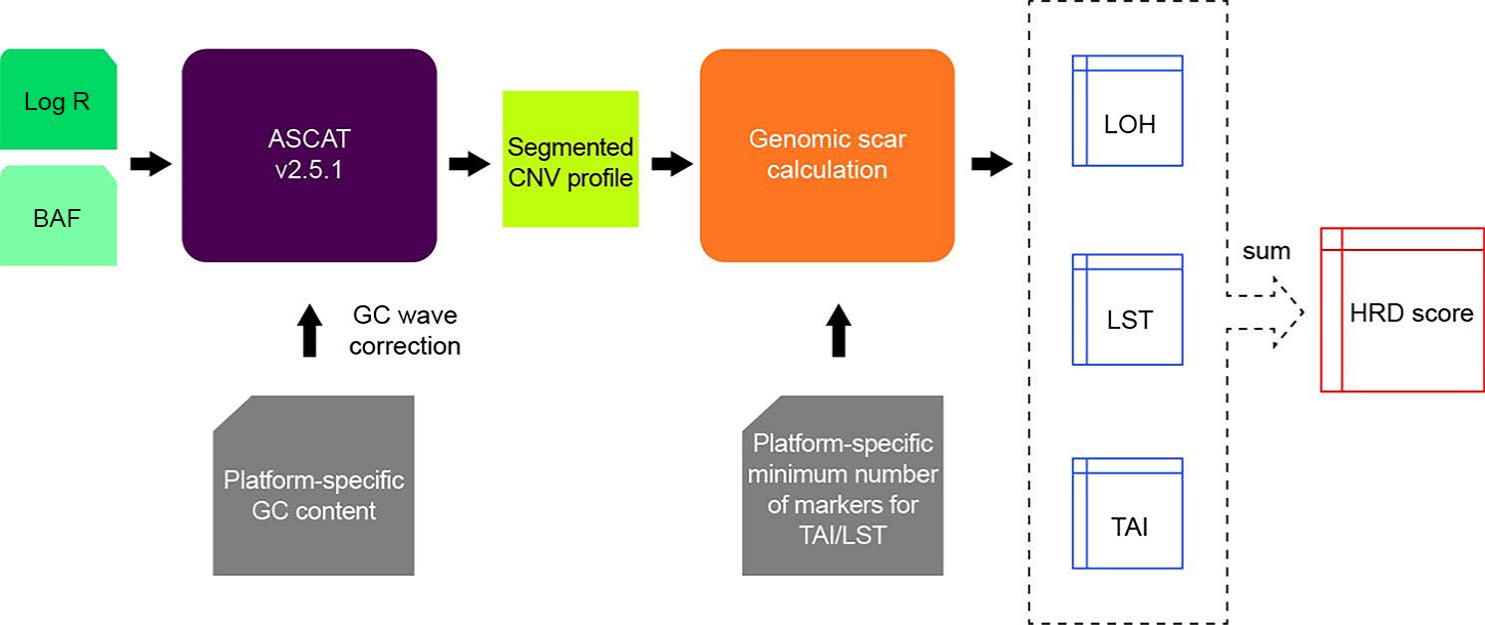
Single-nucleotide polymorphism array data processing diagram. CNV, copy number variation; HRD, homologous recombination deficiency; LOH, loss of heterozygosity; LST, large-scale state transition; TAI, telomeric-allelic imbalance number

GC content is known to affect hybridization yield for Illumina sequencing, resulting in artifactual variations in the inferred copy number across the genome [21]. In our work, platform-specific (OncoScan or Infinium CytoSNP-850K) GC content files were first generated and input to ASCAT (version 2.5.1) for GC correction [22]. ASCAT was then used to evaluate copy number variation (CNV) and LOH (Fig. 1) using log R ratio and B-allele frequency of 205,647 autosomal markers for OncoScan and 789,872 autosomal markers for Infinium CytoSNP-850K, with GC wave correction [23]. Genomic metrics were further generated with default parameters using previously reported algorithms [24]. The aggregate HRD metric was the sum of the three components (LOH, TAI, and LST). LOH was defined as the number of regions longer than 15 megabases (Mb) but shorter than the whole chromosome, with a loss of one normal copy of a gene or a group of genes [25]. TAI was defined as the number of regions with allelic imbalance that extended to one of the subtelomeres but did not cross the centromere [26]. LST was defined as the number of chromosomal break points between adjacent regions of at least 10 Mb after filtering out regions shorter than 3 Mb [27]. Individual LOH, TAI, and LST scores were not available.

The associations between genomic metrics that use *BRCA*m status (defined as deleterious or suspected deleterious mutation) and the clinically validated HRD status test (myChoice CDx) metric (dichotomized at clinical cut-off) were calculated using the area under the receiver operating characteristic (AUROC) curve [28]. AUROC is a descriptive analysis; an AUROC value of < 0.5 is indicative of a negative relationship with the presence of a *BRCA*m, whereas an AUROC value of > 0.5 is indicative of a positive relationship with the presence of a *BRCA*m when the confidence intervals (CIs) in either case do not overlap 0.5. Correlations between continuous metrics were assessed using Spearman and Pearson rank correlation coefficients. CIs of AUROC and correlation coefficients were estimated by the R package pROC (version 1.13.0) and psychometric (version 2.2), respectively. Due to the small sample size of biomarker positive samples (*BRCA*m or HRD positive) in our dataset, area under the precision-recall curve (AUPRC) was also calculated using R package (version 0.11.2). The proportion of biomarker positive samples was taken as baseline value for AUPRC rather than 0.5 for AUROC.

The analytical parameters used in this study can be implemented by individual laboratories using a publicly available algorithm to evaluate HRD locally [23, 24].

### Downsampling

To investigate the relationship between the number of SNP markers and HRD assessment, downsampling of SNP markers was performed to prespecified proportions: 0.25%, 0.5%, 1%, 2.5%, 5%, 10%, 20%, 30%, 40%, 50%, 60%, 70%, 80%, and 90%. Downsampling was conducted randomly 10 times for each proportion, and the median was calculated to represent the HRD score for the downsampling proportion.

## Results

### Clinical tumor samples

Pretreatment archival tumor samples from 126 patients across 20 different tumor types were included in the analysis (Supplementary Table S1, Supplementary Table S2). Of these, CNV segmentation and genomic metrics were successfully calculated for 120 OncoScan, 106 Infinium CytoSNP-850K, and 126 clinically validated test samples.

### Association between genomic metrics per OncoScan or Infinium CytoSNP-850K and *BRCA*m status

**Fig. 2:**
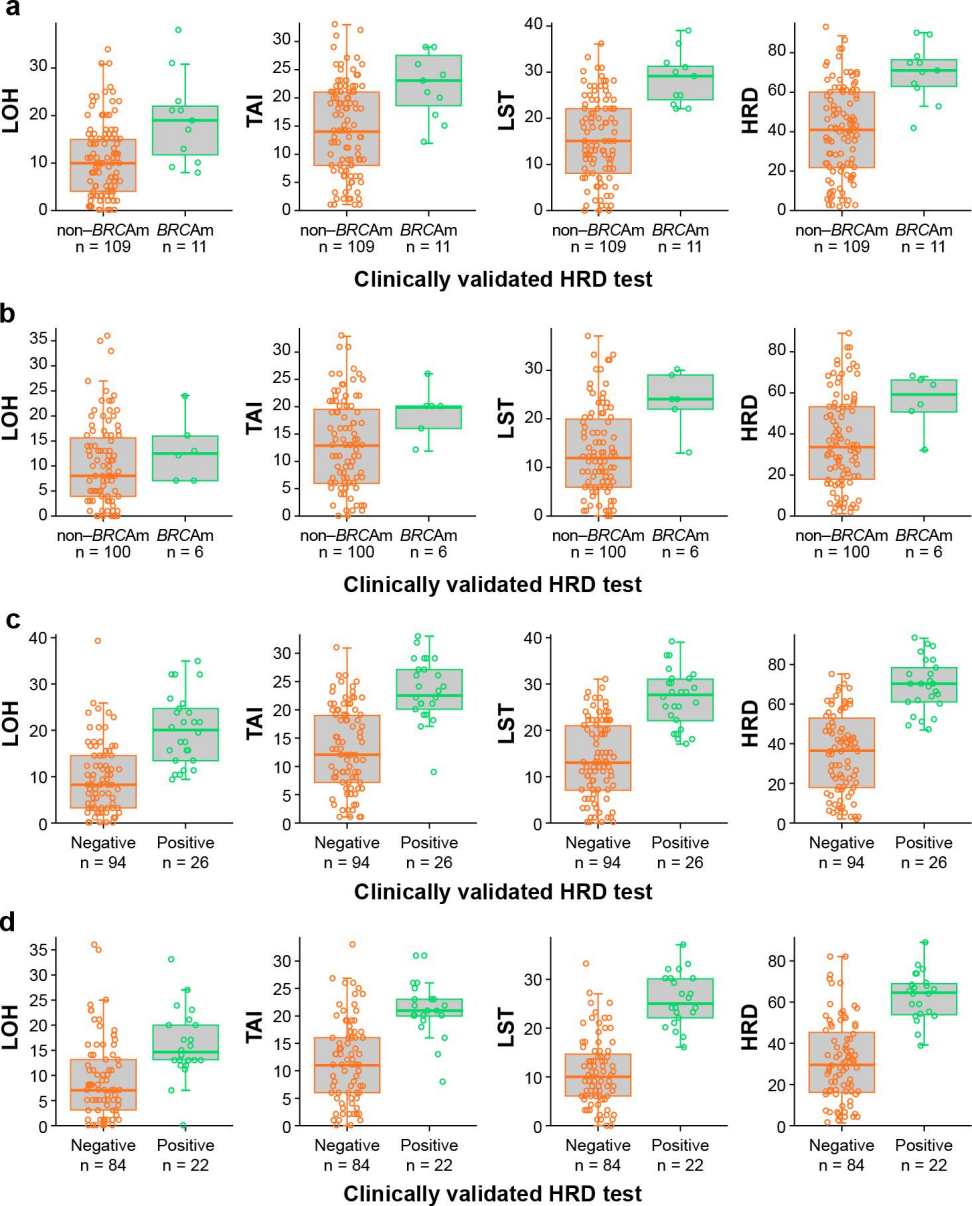
Patient-level genomic metrics by *BRCA*m or HRD status with the clinically validated HRD test. (A) OncoScan and (B) Infinium CytoSNP-850K by *BRCA*m status, and (C) OncoScan and (D) Infinium CytoSNP-850K by HRD status. Orange indicates non–*BRCA*m tumors or HRD negative tumors by the clinically validated HRD test; green indicates deleterious *BRCA*m tumors or tumors deemed HRD positive by the clinically validated HRD test. *BRCA*m, mutation in *BRCA1, BRCA2*, or both; HRD, homologous recombination deficiency; LOH, loss of heterozygosity; LST, large-scale state transition; TAI, telomeric-allelic imbalance number

Of the 120 patients included in the OncoScan analysis, 109 had non–*BRCA*m tumors, and 11 had *BRCA*m tumors as determined by the clinically validated HRD test. Patient-level LOH, TAI, LST, and HRD scores without downsampling per OncoScan by *BRCA*m status are shown in Fig. 2A. The medians of the genomic metrics assessed using OncoScan were all numerically higher in patients with *BRCA*m versus non–*BRCA*m tumors.

Of the 106 patients included in the Infinium CytoSNP-850K analysis, 100 had non–*BRCA*m tumors and six had *BRCA*m tumors as determined by the clinically validated HRD test. Patient-level LOH, TAI, LST, and HRD scores without downsampling per Infinium CytoSNP-850K by *BRCA*m status are shown in Fig. 2B. The medians of the genomic metrics assessed using Infinium CytoSNP-850K were all numerically higher in patients with *BRCA*m versus non–*BRCA*m tumors.

Good association was seen between genomic metrics as a continuous variable and with the presence of a *BRCA*m (determined by the clinically validated HRD test) when assessed using SNP genotyping arrays (Table 1, Supplementary Table S3). When assessed using OncoScan, the AUROC of HRD score versus the presence of a *BRCA*m was 0.87 (95% CI 0.77–0.97); the AUROC of the individual HRD component score versus the presence of a *BRCA*m was 0.76 (95% CI 0.62–0.89) for LOH, 0.78 (95% CI 0.65–0.90) for TAI, and 0.89 (95% CI 0.82–0.96) for LST. When assessed using Infinium CytoSNP-850K, the AUROC of HRD score versus the presence of a *BRCA*m was 0.75 (95% CI 0.61–0.89); the AUROC of the individual HRD components versus the presence of a *BRCA*m was 0.64 (95% CI 0.46–0.82) for LOH, 0.72 (95% CI 0.57–0.87) for TAI, and 0.83 (95% CI 0.70–0.95) for LST.

**Table 1:** AUROC of genomic metrics as a continuous variable

AUROC (95% CI)	OncoScan *n* = 120	Infinium CytoSNP-850K *n* = 106
**With deleterious ** ***BRCA*** **m**		
LOH versus *BRCA*m	0.76 (0.62–0.89)	0.64 (0.46–0.82)
TAI versus *BRCA*m	0.78 (0.65–0.90)	0.72 (0.57–0.87)
LST versus *BRCA*m	0.89 (0.82–0.96)	0.83 (0.70–0.95)
HRD^a^ versus *BRCA*m	0.87 (0.77–0.97)	0.75 (0.61–0.89)
**With the clinically validated HRD test (cut-off, 42)**		
LOH versus clinically validated HRD test	0.85 (0.77–0.92)	0.78 (0.67–0.88)
TAI versus clinically validated HRD test	0.86 (0.79–0.93)	0.86 (0.78–0.93)
LST versus clinically validated HRD test	0.89 (0.82–0.95)	0.95 (0.90–0.99)
HRD^a^ versus clinically validated HRD test	0.92 (0.87–0.97)	0.91 (0.85–0.96)

^a^HRD is the sum of LOH, TAI, and LST

Abbreviations: AUROC, area under the receiver operating characteristic; *BRCA*m, mutation in *BRCA1, BRCA2*, or both; CI, confidence interval; HRD, homologous recombination deficiency; LOH, loss of heterozygosity; LST, large-scale state transition; TAI, telomeric-allelic imbalance number

### Association between genomic metrics per OncoScan or Infinium CytoSNP-850K and HRD status as determined by the clinically validated test

Of the 120 patient samples included in the OncoScan analysis, 94 were HRD negative, and 26 were HRD positive by the clinically validated HRD test (cut-off 42). Patient-level LOH, TAI, LST, and HRD scores without downsampling per OncoScan by HRD status using the clinically validated HRD test are shown in Fig. 2C.

Of the 106 patient samples included in the Infinium CytoSNP-850K analysis, 84 were HRD negative, and 22 were HRD positive by the clinically validated HRD test. Patient-level LOH, TAI, LST, and HRD scores per Infinium CytoSNP-850K by HRD status using the clinically validated HRD test are shown in Fig. 2D.

It is well known that GC content can skew analyses, and thus, we performed an analysis with GC correction. The genomic metric as a continuous variable with the genotyping assays with GC correction demonstrated good association with the clinically validated test at a cut-off of 42 (Table 1, Supplementary Table S3). When assessed using OncoScan, the AUROC of HRD score versus the clinically validated test (cut-off 42) was 0.92 (95% CI 0.87–0.97); the AUROC of individual HRD component score versus the clinically validated test was 0.85 (95% CI 0.77–0.92) for LOH, 0.86 (95% CI 0.79–0.93) for TAI, and 0.89 (95% CI 0.82–0.95) for LST. When assessed using Infinium CytoSNP-850K, the AUROC of HRD score versus the clinically validated test (cut-off 42) was 0.91 (95% CI 0.85–0.96); the AUROC of individual HRD component score versus the clinically validated test was 0.78 (95% CI 0.67–0.88) for LOH, 0.86 (95% CI 0.78–0.93) for TAI, and 0.95 (95% CI 0.90–0.99) for LST.

### Correlation between genomic metrics as a continuous variable per OncoScan or Infinium CytoSNP-850K and the clinically validated HRD test

**Fig. 3:**
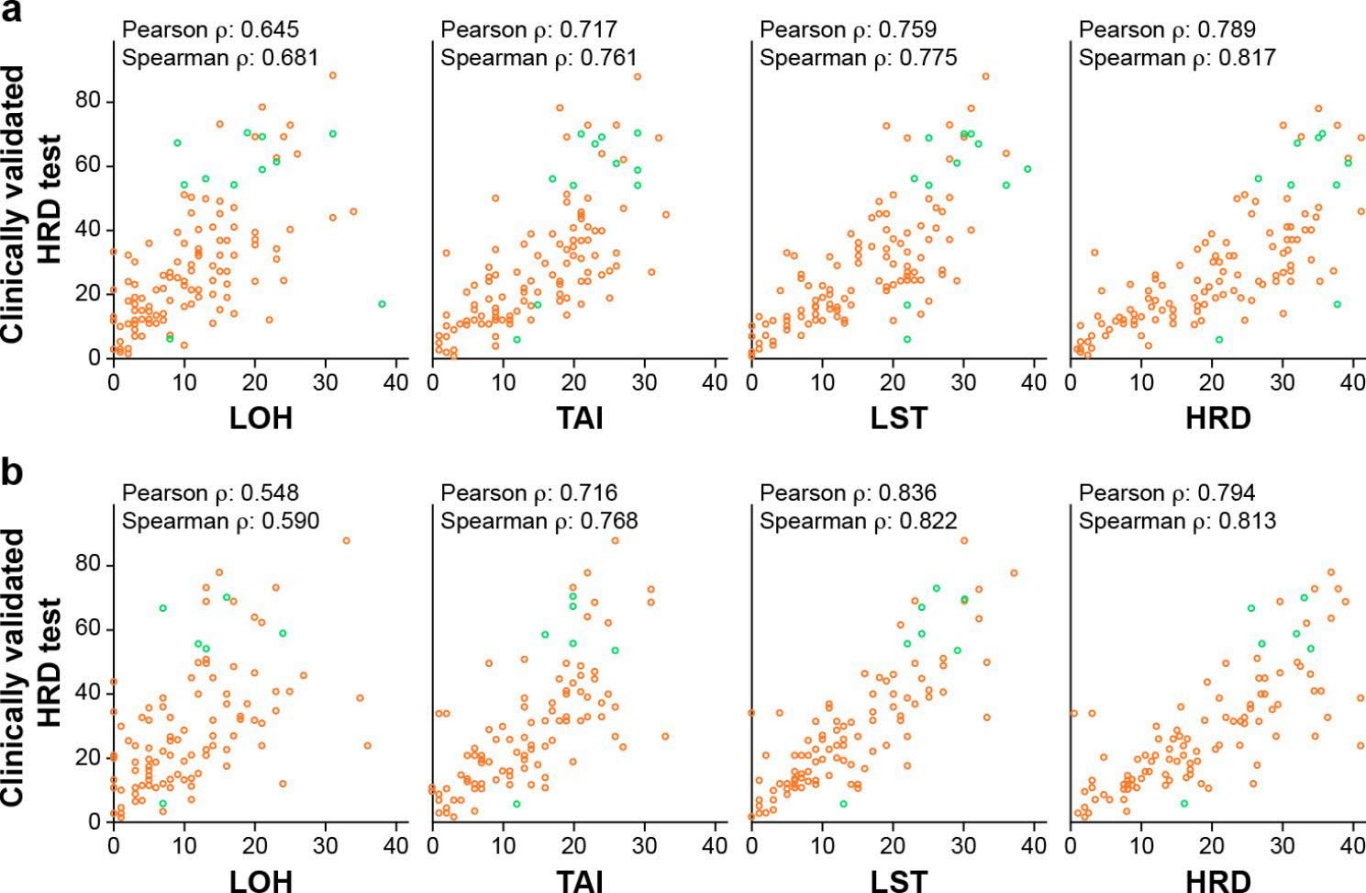
Correlation with GC correction between genomic metrics and the clinically validated HRD test. (A) OncoScan. (B) Infinium CytoSNP-850K. Orange circles indicate non–*BRCA*m tumors; green circles indicate deleterious *BRCA*m tumors. *BRCA*m, mutation in *BRCA1, BRCA2*, or both; HRD, homologous recombination deficiency; LOH, loss of heterozygosity; LST, large-scale state transition; TAI, telomeric-allelic imbalance number

The genomic metric as a continuous variable with GC correction and without downsampling showed good correlation between the SNP genotyping assays and the clinically validated HRD test metric (Spearman ρ: OncoScan, 0.82 [95% CI 0.75–0.87] and Pearson ρ: OncoScan, 0.79, Fig. 3A; Infinium CytoSNP-850K, 0.81 [95% CI 0.74–0.87] and 0.79, Fig. 3B). Without GC correction, the correlation was poorer, particularly for HRD score derived on the Infinium CytoSNP-850K platform (Spearman ρ: OncoScan, 0.80 [95% CI 0.71–0.87]; Infinium CytoSNP-850K, 0.58 [95% CI 0.42–0.70]) (Supplementary Fig. S1). Among individual scores, LST had the highest correlation with HRD status for both OncoScan and Infinium CytoSNP-850K, consistent with the previously reported selection of LST as HRD score [29].

**Fig. 4:**
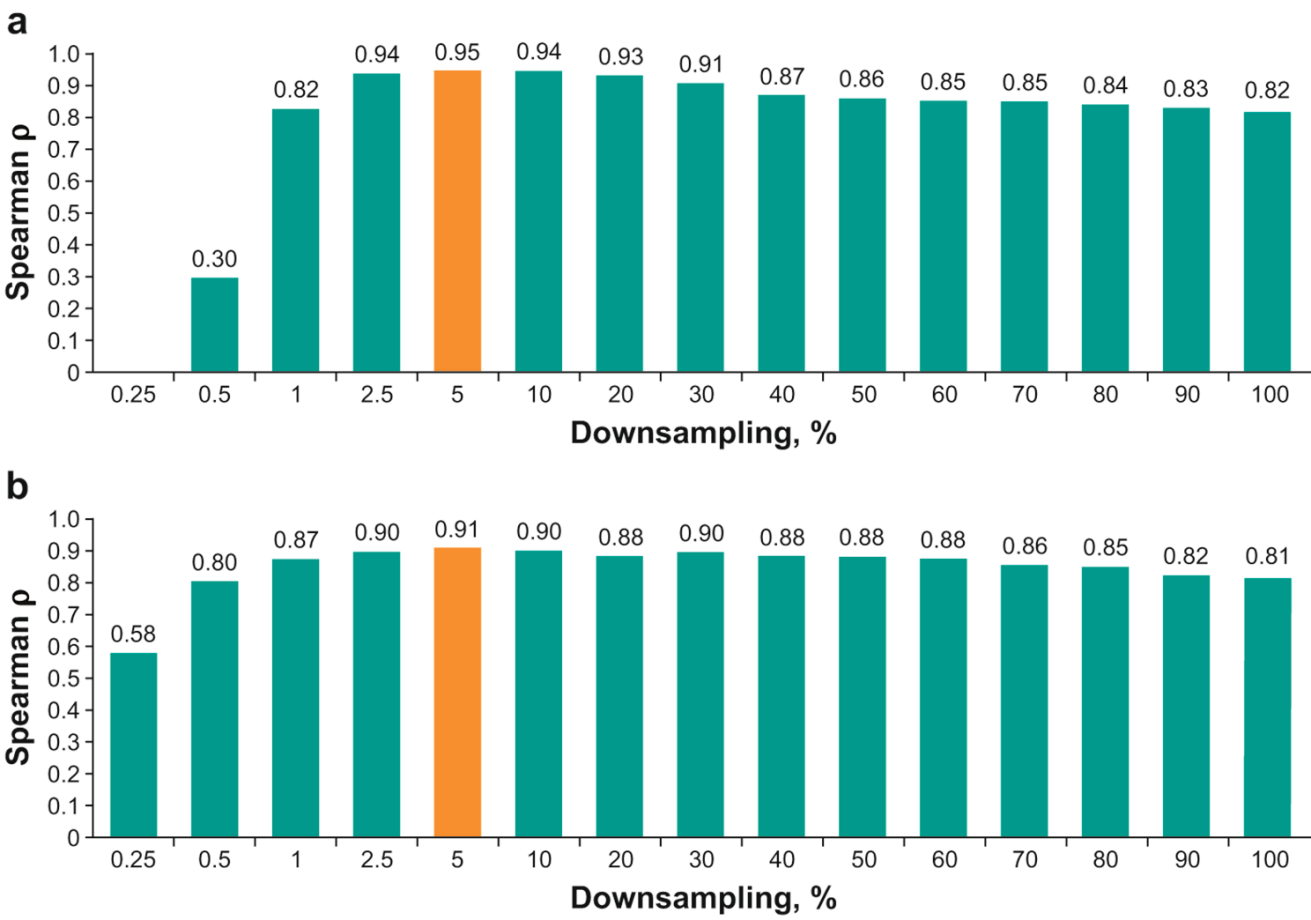
Correlation between HRD sum score by SNP genotyping assays and the clinically validated HRD test following SNP downsampling. (A) OncoScan. (B) Infinium CytoSNP-850K. HRD, homologous recombination deficiency; SNP, single-nucleotide polymorphism

Another metric that can affect the accuracy of determining contiguous regions of genomic LOH is the density of SNPs; the use of too few SNPs may underestimate LOH by not using information from enough loci, whereas using too many SNPs may overestimate the LOH by fitting noisy neighboring loci. We evaluated the effect of downsampling on the accuracy of calculating the genomic metrics; HRD sum score concordance between the SNP array platforms and the clinically validated HRD test was thus further optimized by SNP downsampling (Fig. 4). The optimal (minimum) SNP downsampling factor (maximum correlation with the clinically validated HRD test while maintaining nearly full ASCAT evaluability) was 5% for both OncoScan (median Spearman ρ 0.95) and Infinium CytoSNP-850K (median Spearman ρ 0.91).

## Discussion

Given that HRD assessment has demonstrated both predictive and prognostic values for patients with advanced solid tumors receiving PARPis, including patients with ovarian cancer, several HRD testing strategies are being developed to identify patients likely to benefit from this treatment [2, 13, 30]. Despite the availability of several clinically validated companion HRD assays [2, 31], a gold standard HRD test has not been uniformly accepted, and currently available HRD tests face several limitations regarding more dynamic and more uniform testing harmonization. SNP-based strategies were the basis of most currently used HRD genomic assays that evaluated copy number, many of which use proprietary scoring methods and predefined cut-offs that have not been fully validated or adopted for use in clinical studies [2]. In the current study, common genomic metrics including HRD score as a continuous variable assessed by SNP genotyping assays (OncoScan; Infinium CytoSNP-850K) showed good correlation with the clinically validated HRD test metric. Notably, GC correction did not show much effect on OncoScan results. Composite scoring based on LOH, TAI, and LST and use of different cut-offs to evaluate HRD status have been assessed in several clinical studies [9, 11, 12, 15, 30, 32]. Such studies investigated the predictive value of various HRD testing strategies and the value of demonstrating clinically significant association between HRD status and survival benefits, particularly in patients with *BRCA*m tumors.

Differing HRD measures and cut-offs also pose an important challenge and require harmonization for guiding treatment decisions. For instance, one of the commonly used HRD tests is thought to use an unweighted sum of TAI, LST, and LOH and a dichotomous threshold using a cut-off of 42 based on a training cohort to determine high or low genomic instability score. Yet another common test uses NGS to determine high or low LOH with a predefined cut-off of ≥ 16%. The clinical utility for which various HRD tests are currently stratified further complicates harmonization. For some, but not all, tests, stratification is based on the test’s respective performance in predicting clinical benefit from PARPis rather than their absolute ability to detect HRD. Recent studies have suggested that combining scores from different tests may enhance HRD testing competence [2].

A key limitation shared by gene panel–based HRD tests and SNP-based arrays is that they provide a snapshot of past mutational processes and may not provide an accurate representation of the current activity of DNA repair mechanisms [2, 33]. None of the currently available DNA sequencing tests have the capability to assess the presence of known mechanisms of clinical resistance to PARPis [2]. Regarding limitations of the current analysis, the study was biased by selection of samples with known success toward DNA yield. In addition, we used samples that were available and which met our need and only reported analytical concordance without any clinical response data to PARPis.

## Conclusion

To our knowledge, our study is the first to demonstrate that there is an optimal number of SNP markers used to calculate HRD metrics that maximizes associations with other HRD-related orthogonal markers, and that number is not the maximum on either platform, but rather a fraction. A possible explanation for these findings may be an abundance of SNP markers that would bring more noise for copy number segmentation, which is a fundamental part of HRD metrics calculation. Our study demonstrated that GC content correction had a significantly different effect on OncoScan and CytoSNP data; it also showed that the correlation between median GC content and log R ratio (a measure of total signal intensity) for each 1-Mb region of genome was much higher for CytoSNP than OncoScan data (Wilcoxon rank test, *P* < 0.01) [34]. This might be due to variable input DNA quantity for the CytoSNP assay (data not shown) compared with the OncoScan assay for which uniform DNA quantity was used, which was concordant with previous reports [35]. Taken together, tests based on commercially available SNP-based platforms may potentially be able to identify most HRD-positive tumors (as defined by clinically approved assays), as demonstrated by the high analytical concordance between OncoScan and CytoSNP-850K and the clinically validated test (> 0.90); however, appropriate clinically relevant cut-offs must be determined.

## Electronic supplementary material

Below is the link to the electronic supplementary material.

Supplementary Material 1

## Data Availability

Merck Sharp & Dohme LLC, a subsidiary of Merck & Co., Inc., Rahway, NJ, USA (MSD), is committed to providing qualified scientific researchers access to anonymized data and clinical study reports from the company’s clinical trials for the purpose of conducting legitimate scientific research. MSD is also obligated to protect the rights and privacy of trial participants and, as such, has a procedure in place for evaluating and fulfilling requests for sharing company clinical trial data with qualified external scientific researchers. The MSD data sharing website (available at: http://engagezone.msd.com/ds_documentation.php) outlines the process and requirements for submitting a data request. Applications will be promptly assessed for completeness and policy compliance. Feasible requests will be reviewed by a committee of MSD subject matter experts to assess the scientific validity of the request and the qualifications of the requestors. In line with data privacy legislation, submitters of approved requests must enter into a standard data-sharing agreement with MSD before data access is granted. Data will be made available for request after product approval in the US and EU or after product development is discontinued. There are circumstances that may prevent MSD from sharing requested data, including country or region-specific regulations. If the request is declined, it will be communicated to the investigator. Access to genetic or exploratory biomarker data requires a detailed, hypothesis-driven statistical analysis plan that is collaboratively developed by the requestor and MSD subject matter experts; after approval of the statistical analysis plan and execution of a data-sharing agreement, MSD will either perform the proposed analyses and share the results with the requestor or will construct biomarker covariates and add them to a file with clinical data that is uploaded to an analysis portal so that the requestor can perform the proposed analyses.

## Abbreviations

AUROC: area under the receiver operating characteristic
*BRCA*m: mutation in *BRCA1, BRCA2*, or both
CNV: copy number variation
DNA: deoxyribonucleic acid
FDA: US Food and Drug Administration
HGSOC: high-grade serous ovarian cancer
HRD: homologous recombination deficiency
LOH: loss of heterozygosity
LST: large-scale state transition
NGS: next-generation sequencing
PARP: poly(adenosine diphosphate-ribose) polymerase
PARPi: poly(adenosine diphosphate-ribose) polymerase inhibitor
SNP: single-nucleotide polymorphism
TAI: telomeric-allelic imbalance number
