# Supplementary material for: Concordance between single-nucleotide polymorphism–based genomic instability assays and a next-generation sequencing–based homologous recombination deficiency test
Source: BMC Cancer. 2022 Dec 14;22:1310. doi: 10.1186/s12885-022-10197-z (PMC9749332; doi:10.1186/s12885-022-10197-z)
Supplement: Supplementary file 1 — Supplementary Material 1 [file 12885_2022_10197_MOESM1_ESM.docx]

**Concordance between single-nucleotide polymorphism–based genomic instability assays and a next-generation sequencing–based homologous recombination deficiency test**

Razvan Cristescu^1*^, Xiao Qiao Liu^2^, Gladys Arreaza^1^, Cai Chen^1^, Andrew Albright^1^, Ping Qiu^1^ & Matthew J. Marton^1^

^1^Merck & Co., Inc., Rahway, NJ, USA; ^2^MSD R&D (China) Co. Ltd, Beijing, China

**Supplementary Information**

**Figure S1** Correlation without GC correction between metrics and a clinically validated HRD test. (**a**) OncoScan and (**b**) Infinium CytoSNP-850K. Orange circles indicate non–BRCAm wild type tumors; green circles indicate deleterious BRCAm tumors.

BRCAm, mutation in *BRCA1* or *BRCA2* or both; HRD, homologous recombination deficiency; LOH, loss of heterozygosity; LST, large-scale state transition; TAI, telomeric-allelic imbalance number.


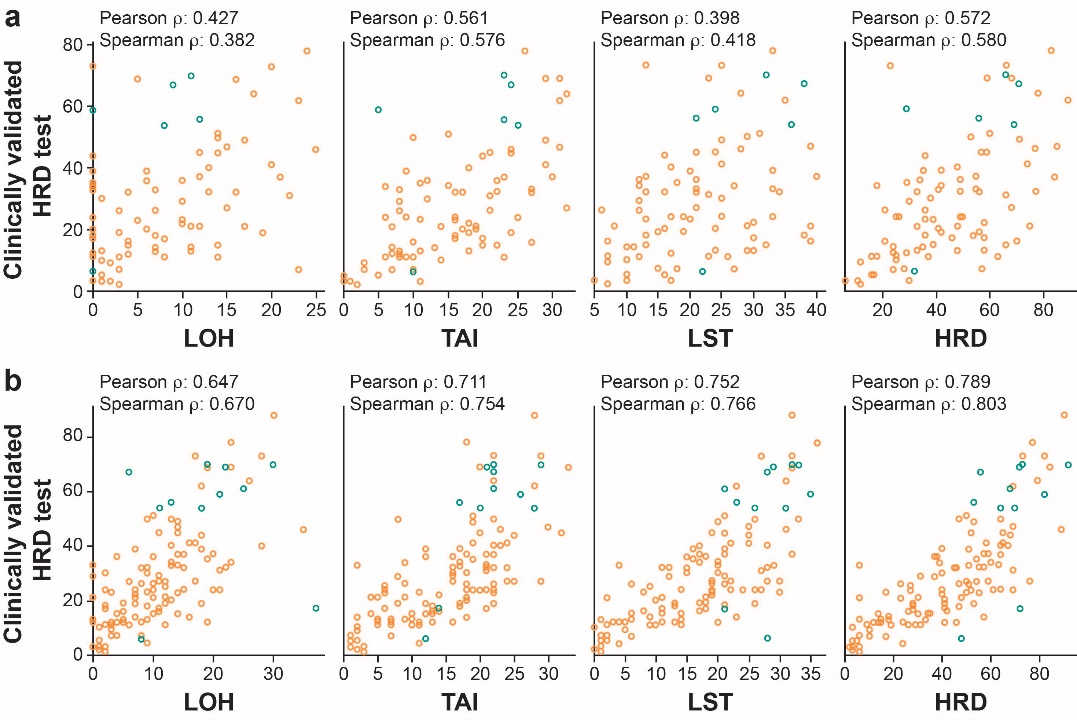


**Table S1 Tumor type distribution for single-nucleotide polymorphism array data in the training set.**


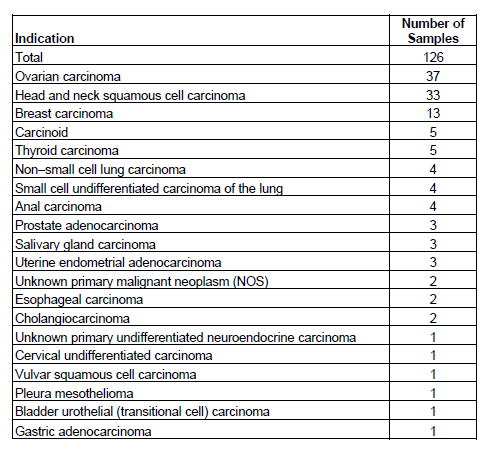


**Table S2 *BRCA*m and HRD status by tumor type.**

*BRCA*m, mutation in *BRCA1*, *BRCA2*, or both; HRD, homologous recombination deficiency.

| **Tumor type** | **Samples, *n*** | **BRCAm, *n*** | **HRD positive , *n*** |
| --- | --- | --- | --- |
| Ovary | 37 | 6 | 14 |
| Head and neck | 33 | 2 | 2 |
| Breast | 13 | 2 | 7 |
| Lung | 9 | 0 | 1 |
| Unknown | 8 | 0 | 1 |
| Thyroid | 5 | 0 | 0 |
| Anus | 4 | 0 | 0 |
| Prostate | 3 | 0 | 2 |
| Salivary gland | 3 | 0 | 0 |
| Endometrium | 2 | 0 | 0 |
| Esophagus | 2 | 0 | 0 |
| Liver | 2 | 1 | 0 |
| Bladder | 1 | 0 | 0 |
| Cervix | 1 | 0 | 0 |
| Skin | 1 | 0 | 0 |
| Stomach | 1 | 0 | 0 |
| Uterus | 1 | 0 | 0 |
| Total | 126 | 11 | 27 |

**Table S3 AUPRC of genomic metrics as a continuous variable.**

^a^The baseline is the equal to the fraction of positive samples.

AUPRC, area under the precision-recall curve; *BRCA*m, mutation in *BRCA1*, *BRCA2*, or both; HRD, homologous recombination deficiency; LOH, loss of heterozygosity; LST, large-scale state transition; TAI, telomeric-allelic imbalance number.

| **AUPRC** | **OncoScan** | **Infinium CytoSNP-850K** |
| --- | --- | --- |
|  | ***n* = 120** | ***n* = 106** |
| **With deleterious *BRCA*m** | | |
| Baseline from *BRCA*m^a^ | 0.09 | 0.06 |
| LOH versus *BRCA*m | 0.29 | 0.08 |
| TAI versus *BRCA*m | 0.23 | 0.09 |
| LST versus *BRCA*m | 0.47 | 0.15 |
| HRD versus *BRCA*m | 0.36 | 0.10 |
| **With a clinically validated HRD test (cut-off: 42)** | | |
| Baseline for clinically validated HRD test^a^ | 0.22 | 0.21 |
| LOH versus clinically validated HRD test | 0.56 | 0.39 |
| TAI versus clinically validated HRD test | 0.64 | 0.50 |
| LST versus clinically validated HRD test | 0.73 | 0.76 |
| HRD versus clinically validated HRD test | 0.79 | 0.61 |
